# An Ultra-Rare Disorder: Case Report on Cerebrotendinous Xanthomatosis

**DOI:** 10.3390/reports8020077

**Published:** 2025-05-22

**Authors:** Mariya Levkova, Mari Hachmeriyan, Margarita Grudkova, Mihael Tsalta-Mladenov, Ara Kaprelyan

**Affiliations:** 1 Department of Medical Genetics, Medical University Varna, Marin Drinov Str. 55, 9000 Varna, Bulgaria; 2 Laboratory of Medical Genetics, St. Marina Hospital, Hristo Smirnenski Blv 1, 9000 Varna, Bulgaria; 3 Department of Neurology and Neuroscience, Medical University Varna, Marin Drinov Str. 55, 9000 Varna, Bulgaria

**Keywords:** cerebrotendinous xanthomatosis, xanthoma, cataract, rare disorder, case report

## Abstract

**Background and Clinical Significance:** Cerebrotendinous xanthomatosis (CTX) is a rare autosomal recessive disorder caused by mutations in the *CYP27A1* gene, leading to impaired bile acid synthesis and systemic cholesterol deposition. The condition presents with a broad spectrum of symptoms affecting multiple organs and systems, including the eyes, central nervous system, tendons, and skeletal muscles. Due to its heterogeneous and often ambiguous clinical manifestations, CTX is frequently misdiagnosed or remains undiagnosed for years. **Case Presentation**: We report the case of a 37-year-old male who was admitted to our university hospital with a long-standing history of progressive muscle weakness in the arms and legs. His medical history revealed bilateral cataract surgery in childhood, cognitive decline, epilepsy, and bilateral round swellings of the Achilles tendons, suspected to be xanthomas. A clinical diagnosis of CTX was established, and sequencing analysis confirmed the presence of a homozygous pathogenic variant in the *CYP27A1* gene. Despite the unavailability of chenodeoxycholic acid (CDCA) therapy in Bulgaria, symptomatic management was provided. **Conclusions**: This case underscores the diagnostic challenges associated with CTX and highlights the prolonged diagnostic journey faced by patients with rare neurogenetic disorders. It also emphasizes the need for increased awareness and early recognition of such conditions to improve patient outcomes.

## 1. Introduction and Clinical Significance

Cerebrotendinous xanthomatosis (CTX, OMIM 213700) is a rare autosomal recessive disorder, classified as ultra-rare due to its extremely low prevalence among various populations. Among Europeans, Americans, and African Americans, the estimated prevalence ranges from approximately 1 in 70,795 to 1 in 233,597 individuals [1]. CTX can be described as an inborn error of metabolism, arising from a defect in the bile acid synthesis pathway caused by a deficiency of the enzyme 27-hydroxylase (*CYP27A1*) [2]. This enzymatic defect leads to the impaired metabolism of cholesterol, causing the abnormal accumulation of cholestanol, a derivative of cholesterol, in various tissues [3].

The primary site of cholestanol accumulation is the central nervous system, leading to significant neurological manifestations. However, CTX also causes a range of non-neurological symptoms, affecting multiple organs and systems. Clinical presentations of CTX often include intellectual disability, progressive neurological decline, cataracts, tendon xanthomas, and an elevated risk of cardiovascular disease, among other symptoms [3]. Given this diverse and sometimes ambiguous clinical picture, CTX is frequently misdiagnosed or undiagnosed for years, especially in general clinical practice, where rare diseases may not be considered as part of differential diagnoses [4,5].

This report details the diagnostic journey of a male patient with CTX, emphasizing the challenges of identifying rare neurogenetic disorders. Notably, this is the first documented case of CTX in Bulgaria, providing a comprehensive clinical description that aims to raise awareness and improve early recognition and diagnosis in our region. Additionally, this case highlights the prolonged and complex diagnostic process that many patients with rare diseases endure, often spanning years due to the rarity of CTX and limited awareness among healthcare providers [5].

## 2. Case Presentation

We present a 37-year-old male admitted to the First Clinic of Neurology, University Hospital “Saint Marina”, Varna, Bulgaria, with long-standing muscle weakness in his arms and legs. Written informed consent was obtained from the patient for the publication of this case report, including the accompanying photographs.

Born prematurely via C-section with a birth weight of 1500 g, he had normal early neurological development but later attended auxiliary school due to mild intellectual disability. At 15, he experienced epileptic seizures managed with valproic acid, remaining seizure-free. At 20, he was diagnosed with bilateral cataracts, surgically corrected. In 2020, he suffered a ruptured Achilles tendon and a leg fracture, after which his walking difficulties worsened, now requiring a wheelchair. He had no behavioral risk factors or family history of genetic diseases. A neurologist suspected multiple sclerosis and referred him for hospitalization.

**Figure 1:**
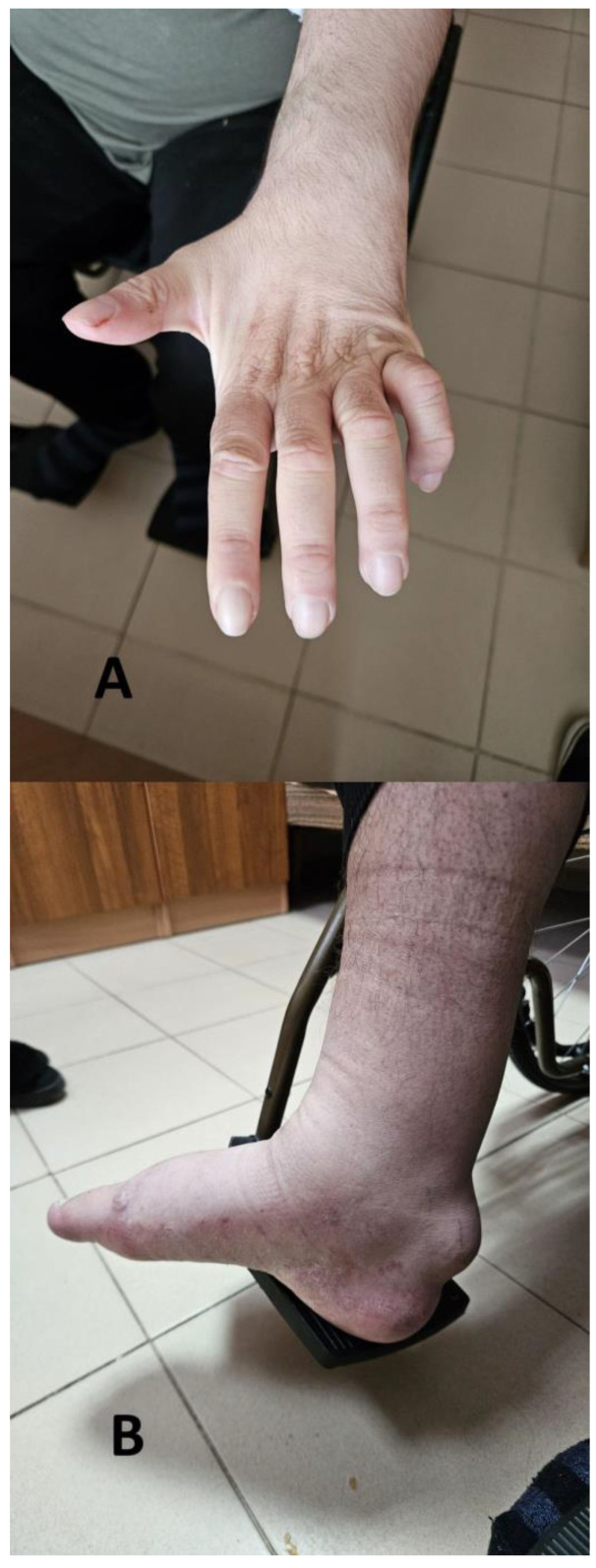
Contractures for the interphalangeal joints of the hands (**A**) and xanthomas on the Achilles tendon of the patient (**B**).

The neurological examination of the patient revealed the following: cranial nerve assessment was normal. Mild dysarthria was present. The motor activity examination revealed with decreased muscle strength for the lower limbs was 2/5, and was more severe for the distal muscle groups of the lower limbs. Muscle tone was decreased for the lower limbs, while there were bilateral muscle contractures for the interphalangeal joints of the hands (Figure 1A). There was quadrihyporeflexia with symmetrical, just elicitable (−3), deep tendon reflexes and no pathological reflexes from the Babinski and Rossolimo groups. Bilateral round formations suspected for xanthomas of the Achilles tendons were observed (Figure 1B). The Finger-to-nose test revealed a bilateral dissymmetry. The levels of cholesterol and triglycerides were within the normal range.

**Figure 2:**
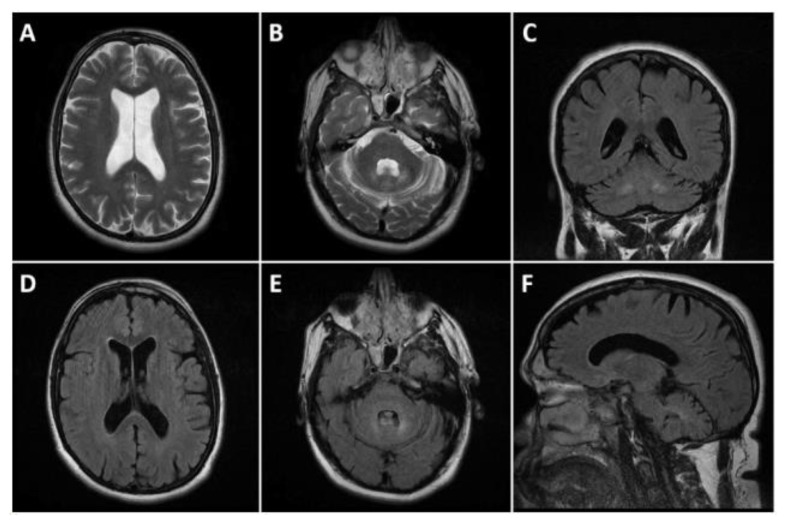
Head MRI presenting cortical atrophy and bilateral changes in the signal intensity in the zones of the dentate nucleus. (**A**,**B**): T2 axials; (**D**,**E**): FLAIR axials; (**C**): FLAIR coronal, and (**F**): FLAIR sagittal. Dilated ventricular system and subarachnoid fluid spaces on the convexity bilaterally (**A**,**D**). Symmetric T2 and FLAIR hyperintensities affecting the dentate nuclei (**B**,**C**,**E**,**F**).

The magnetic resonance imaging (MRI) of the head showed dilatation of the cerebral ventricles, cortical atrophy, and bilateral changes in the signal intensity in the zones of the nuclei dentati (increased signal of T2 and T2 FLAIR images, without significant changes in diffusion and gradient images). The ventricular system, cerebral cistern, and subarachnoid fluid spaces on convex were dilated (Figure 2). The electroencephalogram (EEG) showed diffuse medium-voltage, and in places high-voltage, bisynchronous theta activity with single spike–wave discharges (SWDs). The T carotid artery duplex scan was normal.

The higher-cognitive-function assessment revealed, for a Mini-Mental State Examination (MMSE), 21/30 points, indicative of mild dementia; for Isaac’s Set Test (IST), 32 points; and for the Instrumental Activities of Daily Living Scale (IADL), 3/8 points. The ophthalmological examination revealed bilateral pseudophakia after the cataract surgeries.

Whole exome sequencing (WES), performed at DanteLabs, Italy, identified a homozygous variant c.646G>C (p.Ala216Pro) in the *CYP27A1* gene, consistent with a diagnosis of CTX. Variant interpretation was supported by the Franklin by Genoox platform [6], which aids in the classification of sequence variants. According to the American College of Medical Genetics and Genomics guidelines [7], this variant was classified as pathogenic based on several criteria, including its absence from population databases (e.g., gnomAD), deleterious predictions from in silico tools, and previously reported associations with CTX in the literature. The patient’s sister was identified as a heterozygous carrier. Although parental testing was not performed, the presence of a heterozygous carrier sibling supports biallelic inheritance of the variant. Nevertheless, in the absence of CNV analysis, the possibility of a heterozygous deletion on one allele (resulting in pseudo-homozygosity) cannot be entirely excluded. However, the patient’s clinical presentation was typical of CTX and the reported finding was considered sufficient to establish the molecular diagnosis in this case.

Following the diagnosis, the patient was admitted to the hospital for re-evaluation and post-genetic counseling. Unfortunately, chenodeoxycholic acid is not available in Bulgaria. Therefore, the patient was prescribed rehabilitation therapy and symptomatic treatment.

## 3. Discussion

CTX is a rare multisystemic disorder that affects the nervous system and other organs [3]. It is caused by pathogenic variants in the *CYP27A1* gene. The variant identified in our patient has been previously reported in the literature in both homozygous and compound heterozygous individuals with CTX [8,9,10].

**Figure 3:**
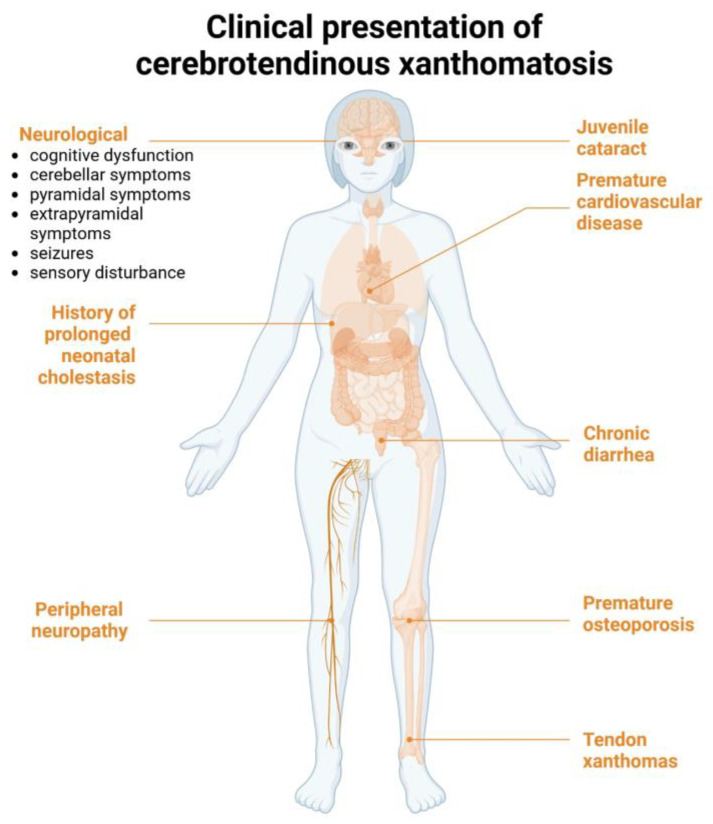
Clinical manifestations of cerebrotendionous xanthomatosis according to Sekijima et al. [11]. Created in Biorender. Mariya Levkova. (2025) https://BioRender.com/.

In 2018, Sekijima et al. proposed a list of diagnostic criteria for CTX [11]. According to these criteria, CTX is characterized by seven main clinical features: tendon xanthoma, progressive neuropsychiatric dysfunction or intellectual disability, juvenile cataract, juvenile coronary artery disease, chronic unexplained diarrhea, juvenile osteoporosis, and prolonged neonatal cholestasis [11]. A confirmed diagnosis requires elevated cholestanol levels, the presence of a pathogenic *CYP27A1* variant in a homozygous or compound heterozygous state, and the exclusion of differential diagnoses such as familial hypercholesterolemia, sitosterolemia, and obstructive biliary tract diseases (Figure 3) [11].

Some studies have noted that cholesterol levels are often normal in most CTX cases, which challenges their inclusion as a diagnostic criterion [12]. In our patient, cholesterol levels were normal, and cholestanol testing was not possible due to its unavailability in our country.

Sekijima et al. defined three diagnostic categories for CTX. The first one is definite, and it requires the presence of at least one main clinical symptom, positive biochemical findings (increased levels of cholestanol), genetic confirmation, and the exclusion of differential diagnoses. The second category is probable—it requires at least one main clinical symptom, biochemical abnormalities, and the exclusion of differential diagnoses. The third category is possible—it requires at least one main clinical symptom and positive biochemical findings [11].

Despite the availability of these diagnostic criteria, CTX is a highly heterogeneous disorder, and its symptoms may vary significantly among affected individuals. For instance, not all patients exhibit tendon xanthomas, a hallmark feature of CTX [3].

Therefore, diagnosing CTX can be challenging, and diagnostic delays are common. Moreover, in some countries, including ours, molecular genetic testing is patient-funded. This may partly explain the lengthy diagnostic journey faced by patients with rare diseases in general, as genetic testing has only become more affordable in recent years. It is recommended to screen all patients under 30 years of age with juvenile cataracts for CTX, especially if they also exhibit chronic diarrhea, tendon xanthomas, or neuropsychiatric symptoms [3,13]. A Brazilian cohort study identified cataracts and xanthomas as the most common non-neurological symptom during the disease course [14].

Neuroimaging findings in CTX are also variable. The most common MRI abnormalities include T2/FLAIR hyperintensity in the cerebellar dentate nuclei and corticospinal tracts, as well as spinal cord atrophy [14]. This was also present in our case. However, the severity of MRI changes does not consistently correlate with the degree of cognitive impairment [15]. Electroencephalogram (EEG) findings, such as diffuse slow waves, have also been reported in CTX patients [16].

The clinical presentation of CTX varies by age. Early symptoms include neonatal-onset cholestasis, infantile-onset diarrhea, and childhood-onset cataracts [2]. Tendon xanthomas typically appear in the second or third decade of life, while neurological dysfunction often manifests in adulthood. Cognitive decline may begin at any age, even in early infancy. However, most patients maintain normal or slightly impaired intellectual function until puberty [5]. In our case, intellectual disability appeared around puberty, as the patient struggled academically in high school.

Behavioral and personality changes are also common in CTX, as illustrated by Chun et al., whose patient exhibited disinhibition, compulsive behavior, apathy, and dietary changes [17]. While intellectual disability is considered a hallmark feature, some patients lack this symptom and instead present with other issues, such as walking difficulties due to painful swelling of the Achilles tendons [5,18].

Our case shares several clinical features commonly observed in cerebrotendinous xanthomatosis (CTX), including early-onset bilateral cataracts, chronic progressive muscle weakness, xanthomas of the Achilles tendons, and intellectual disability. These manifestations are consistent with typical presentations of CTX reported in the literature [11]. However, certain hallmark features such as chronic diarrhea, frequently noted in early stages of the disease, were absent in our patient.

All of the above-described various clinical presentations could explain why the average delay between symptom onset and diagnosis was 16.46 years in a large Brazilian cohort of patients with CTX [14]. In our case, the diagnosis was made 17 years after bilateral cataract surgery, despite juvenile cataracts being a hallmark of CTX. The patient was initially referred with suspected multiple sclerosis, with other frequent misdiagnoses including hereditary spastic paraplegia, hereditary ataxia, and intellectual disability of unknown origin [14]. Interestingly, epilepsy can also be an early manifestation of CTX [18]. Our patient developed epilepsy during adolescence, which may have been part of the initial clinical presentation.

Treatment with chenodeoxycholic acid (CDCA) is the standard of care for CTX. The mechanism of action involves inhibiting the bile acid synthesis pathway, thus decreasing the levels of cholestanol and improving the clinical presentation [2]. The unavailability of CDCA in several countries, including ours, reflects a common challenge associated with the global distribution of orphan drugs. Medications indicated for rare disorders often face limited market incentives, leading to supply constraints and regional disparities in access. This underscores the need for harmonized regulatory frameworks and incentivized production models to improve the availability of essential therapies for rare disease populations [19]. Greater clinical awareness of CTX is also vital, as early recognition and diagnosis greatly influence outcomes. Although CTX is treatable, timely intervention is crucial to prevent irreversible neurological symptoms, halt disease progression, and improve prognosis [2].

## 4. Conclusions

Cerebrotendinous xanthomatosis is a rare genetic disorder with a highly variable phenotype, making early diagnosis challenging. Bilateral cataracts and tendon xanthomas are common, distinguishing features that aid diagnosis. This case underscores the need for heightened clinical awareness in evaluating rare neurological disorders, as prompt diagnosis and early intervention can significantly alter disease trajectory.

## Data Availability

The original contributions presented in this study are included in the article. Further inquiries can be directed to the corresponding author.

## Abbreviations

The following abbreviations are used in this manuscript:

CTX	Cerebrotendinous xanthomatosis
*CYP27A1*	enzyme 27-hydroxylase
MRI	magnetic resonance imaging
EEG	electroencephalogram
SWDs	single spike–wave discharges
MMSE	Mini-Mental State Examination
IADL	Instrumental Activities of Daily Living Scale
CDCA	chenodeoxycholic acid
CNV	copy number variant
